# A Novel tRF, HCETSR, Derived From tRNA‐Glu/TTC, Inhibits HCC Malignancy by Regulating the SPBTN1‐catenin Complex Axis

**DOI:** 10.1002/advs.202415229

**Published:** 2025-02-08

**Authors:** Tao Rui, Kangbei Zhu, Zonglei Mao, Jiaping Wu, Yi Pan, Qianwei Ye, Cong Chen, Aizhai Xiang, Jufeng Guo, Ning Tang, Jing Zhang, Shusen Zheng, Jian Liu, Xiao Xu

**Affiliations:** ^1^ Department of Surgery Affiliated Hangzhou First People's Hospital School of Medicine Westlake University Hangzhou 310003 China; ^2^ The Center for Integrated Oncology and Precision Medicine Affiliated Hangzhou First People's Hospital Zhejiang University School of Medicine Hangzhou 310003 China; ^3^ Department of Surgery Collaborative Innovation Center for the Diagnosis and Treatment of Infectious Diseases the First Affiliated Hospital Zhejiang University School of Medicine Zhejiang University Hangzhou Hangzhou 310003 China; ^4^ School of Clinical Medicine Hangzhou Medical College Hangzhou 310059 China; ^5^ Institute of Translational Medicine Zhejiang University Hangzhou 310000 China; ^6^ NHC Key Laboratory of Combined Multi‐Organ Transplantation Institute of Organ Transplantation Zhejiang University Hangzhou 310003 China

**Keywords:** catenin complex, Dicer1, spliceosome, SPTBN1, tRNA‐derived fragment

## Abstract

tRNA‐derived fragments (tRFs), a novel class of small non‐coding RNAs cleaved from transfer RNAs, have been implicated in tumor regulation. In this study, the role of a specific tRF, HCETSR is investigated, which is significantly downregulated in hepatocellular carcinoma (HCC) and correlates with advanced tumor burden and higher HCC mortality. Functional analyses revealed that HCETSR inhibits HCC malignancy and serves as an independent predictor of poor prognosis. Mechanistically, a novel SPTBN1/catenin complex axis regulated by HCETSR is identified. HCETSR binds to a critical domain of SPTBN1, disrupting its interaction with the catenin complex (comprising β‐catenin, α‐catenin, and P120‐catenin), and facilitates the transfer of the catenin complex from the cell membrane to the nucleus. Specifically, HCETSR decreases the proteasomal degradation of β‐catenin and inhibits the synthesis of nascent β‐catenin. Furthermore, HCETSR suppresses the transcriptional activity of LEF1 through P120‐catenin rather than α‐catenin, thereby reducing β‐catenin's influence on LEF1 activity. It is demonstrated that HCETSR is spliced from tRNA‐Glu/TTC. The biogenesis of HCETSR and tRNA‐Glu/TTC is regulated by the spliceosome and Dicer1. In conclusion, These findings suggest that HCETSR, derived from tRNA‐Glu/TTC, inhibits HCC malignancy via modulation of the SPTBN1/catenin axis and may represent a promising prognostic marker and therapeutic strategy for HCC.

## Graphical abstract

HCETSR, derived from tRNA‐Glu/TTC, binds to SPTBN1, promoting the dissociation of SPTBN1 from the catenin complex and facilitating the translocation of the catenin complex from the cell membrane to the nucleus. Meanwhile, HCETSR reduces proteasomal degradation of β‐catenin and synthesis of nascent β‐catenin. This ultimately inhibits LEF1 transcription factor activity and the hepatocellular carcinoma malignancy.

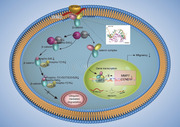

## 1 Introduction

Hepatocellular carcinoma (HCC) is one of the most prevalent malignant tumors, characterized by a poor prognosis.^[^ ^1^ ^]^ The incidence rate of HCC is increasing more rapidly than other cancers, necessitating significant attention.^[^ ^2^ ^]^ Over the past few decades, HCC mortality has risen among both women and men. Surgical intervention remains a critical treatment modality for HCC.^[^ ^3^ ^]^ Despite advancements in treatment, including targeted drug therapy, local ablation, transcatheter arterial chemoembolization, and liver transplantation, the prognosis for HCC continues to be dire.^[^ ^4^, ^5^, ^6^, ^7^ ^]^ Therefore, a deeper understanding of the pathogenesis and the identification of potential therapeutic targets for HCC are imperative.

Transfer RNA⁃derived small RNAs (tsRNAs) are a novel class of small non‐coding RNAs, typically 18–34 nucleotides in length. tsRNAs are generated from specific cleavage of mature or precursor tRNAs (pre‐tRNA),^[^ ^8^ ^]^ a process influenced by stress conditions such as oxidative stress, starvation, and heat shock.^[^ ^9^, ^10^, ^11^ ^]^ Over the past two decades, studies have proposed various classifications for tsRNA, including tRNA‐derived fragments (tRFs) and tRNA halves (tiRNAs).^[^ ^12^, ^13^ ^]^ Different types of tRFs have been shown to have a variety of biological functions, including regulation of translational efficiency, cell viability, RNA degradations, or RNA stability.^[^ ^14^ ^]^ Additionally, they have been implicated in the development of various disease, including metabolic disorder, reproductive defects, cardiac disease, and viral infections.^[^ ^15^, ^16^, ^17^, ^18^ ^]^ Dysregulation of tRFs is highly associated with tumorigenesis and tumor progression.^[^ ^19^ ^]^ Zhou Y et al. discovered that gly‐tRF plays tumor‐promoting role in HCC by targeting NDFIP2 and activating the AKT signaling pathway.^[^ ^20^ ^]^ However, research on the role of tRFs in HCC is limited. Understanding the processes and mechanisms of tRFs may provide potential strategies or targets for HCC therapy.

The cytoskeleton system is a complex network containing many elements that control signaling pathways.^[^ ^21^ ^]^ β‐catenin, α‐catenin, and P120‐catenin are crucial components that contribute to cell‐cell adhesion via the actin cytoskeleton.^[^ ^22^, ^23^ ^]^ Among them, β‐catenin is the most well‐known molecule. It acts as a linker between the transmembrane protein E‐cadherin and the actin cytoskeleton, thereby facilitating cell‐cell adhesion and maintaining tissue integrity.^[^ ^24^ ^]^ Additional, β‐catenin is an activator of the canonical Wnt signaling pathway.^[^ ^25^ ^]^ Upon Wnt pathway activation, β‐catenin translocates to the nucleus and binds to LEF1 transcription factor, regulating the expression of target genes such as MMP7 and CCDN1.^[^ ^26^, ^27^, ^28^ ^]^ Interestingly, β‐catenin has been shown to bind directly to α‐catenin.^[^ ^29^ ^]^ P120‐catenin can control the activity of Kaiso, thereby influencing its interaction with LEF1 and β‐catenin.^[^ ^30^ ^]^ However, it is unknown whether β‐catenin can form a complex with P120‐catenin and α‐catenin.

Spectrin beta, non‐erythrocytic 1 (SPTBN1), a member of the spectrin protein family located on the medial membrane of the cell, is a vital component of the spectrin‐actin cytoskeleton that dynamically remodels cellular integrity, viscoelastic properties, and cell motility in response to cellular signaling or stress.^[^ ^31^, ^32^ ^]^ The spectrin‐based cytoskeleton is initially discovered to be essential for the mechanical stability and elasticity of red blood cells.^[^ ^33^ ^]^ The actin‐binding domains (ABDs) of SPTBN1 consist of calponin homology (CH) domains, which enable SPTBN1 to interact with F‐actin.^[^ ^34^ ^]^ SPTBN1 is associated with various diseases, such as neurological disorders, osteoporosis, and hearing ability.^[^ ^35^, ^36^, ^37^ ^]^ Disorders of SPTBN1 affect the occurrence, progression, and metastasis of cancer by influencing multiple cancer‐related signal pathways.^[^ ^38^ ^]^ In HCC, SPTBN1 stimulates autophagy via SETD7‐mediated YAP Methylation.^[^ ^39^ ^]^ Zhi X et al. also demonstrated that SPTBN1 suppresses HCC progression by inhibiting Wnt signaling l.^[^ ^40^ ^]^ However, the correlations between SPTBN1 and other cytoskeleton components in cancer remain unclear.

In this study, we systematically investigated a novel tRF, HCETSR, which inhibits the metastasis of HCC. We uncovered the regulation of HCETSR on SPTBN1/catenin complex axis. HCETSR interrupts the correlation between SPTBN1 and the catenin complex, regulates the degradation of β‐catenin at the proteasome, decreases the synthesis of nascent β‐catenin, and ultimately influences the LEF1 factor.

## 2 Results

### 2.1 Identification and Validation of HCC‐Associated tsRNA

**Figure 1:**
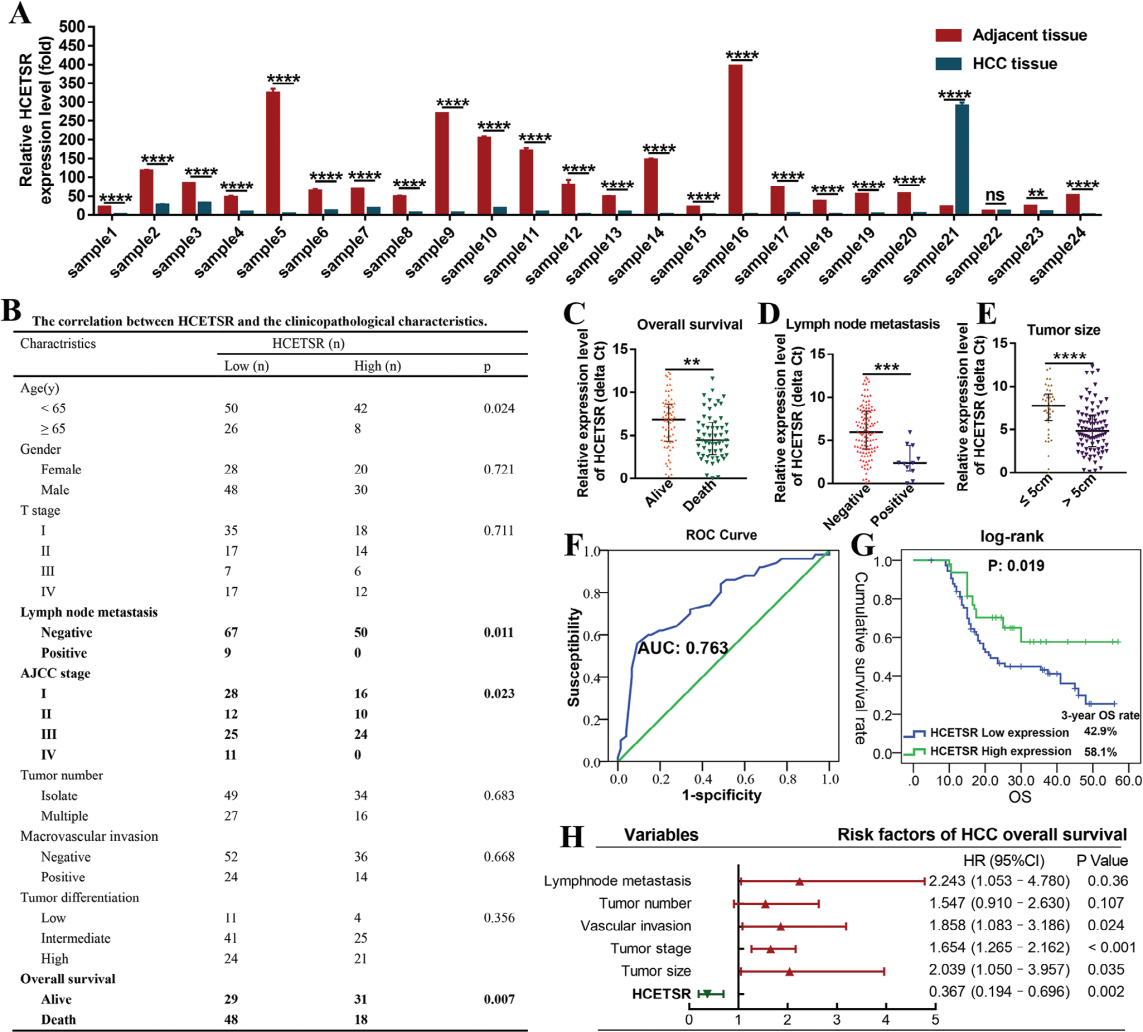
HCETSR is negatively correlated with HCC tumor burden and mortality. A) qRT‐PCR shows that HCETSR is downregulated in HCC tissues compared to the adjacent non‐tumor tissues (24 pairs). B) Chi‐square test indicates the correlation between HCETSR and clinicopathological characteristics, based on 126 HCC samples. C–E) Low HCETSR expression in HCC is associated with poorer overall survival (C), lymph node metastasis (D) and larger tumor size (E). F) ROC curve analysis reveals that HCETSR expression improves the prediction of HCC mortality. G) Kaplan–Meier survival analysis with log‐rank test confirms that low tumor HCETSR expression distinguishes worse HCC overall survival. H) Cox proportional hazards model identifies low HCETSR expression as an independent risk factor in HCC. RT‐qPCR data are presented as mean ± SD from three individual experiments. ^**^ *p* < 0.01, ^***^ *p* < 0.001, and ^****^ *p* < 0.0001. ns, nonsignificant; AUC, area under curve; OS, overall survival; HR, hazard ratio; 95% CI: 95% confidence interval.

To systematically investigate specific tsRNAs that were dysregulated in HCC, we performed small RNA sequencing and constructed tsRNA libraries in HCC samples. Among the differentially expressed tsRNA profile, tRF‐31‐86V8WPMN1E8Y0 (sequence: 5′‐TCCCATATGGTCTAGCGGTTAGGATTCCTGG‐3′) showed the most significant differences (Appendix SA Figure S1, Supporting Information). According to the MINTbase v2.0 database, tRF‐31‐86V8WPMN1E8Y0 functions as 5′‐tRF, potentially spliced from 5′end of tRNA‐Glu/TTC (Appendix SA Figure S2, Supporting Information). Using PCR and Sanger sequencing, we confirmed that the sequence of tRF‐31‐86V8WPMN1E8Y0 was consistent with the expression profile observed (Appendix SA Figure S3, Supporting Information). To verify the expression level of tRF‐31‐86V8WPMN1E8Y0 in HCC, we randomly selected 24 paired of HCC tissues and their corresponding adjacent liver tissues. The results demonstrated that tRF‐31‐86V8WPMN1E8Y0 was significantly downregulated in HCC (**Figure** **1**A). Specifically, 22 out of 24 pairs showed decreased expression, 1 pair remained unchanged, and 1 pair exhibited increased expression. The largest ΔCt value exceeded 8 in one sample. These results confirmed that tRF‐31‐86V8WPMN1E8Y0 was markedly downregulated in HCC. We designated this uncharacterized tsRNA as hepatocellular carcinoma‐effectively‐associated tsRNA (HCETSR).

### 2.2 Low Expression of HCETSR Correlates with HCC Tumor Burden and Independently Predicts HCC Mortality

We analyzed the correlation between HCC features and tumor HCETSR expression in 126 HCC samples. The chi‐square test revealed a negative correlation between HCETSR levels and both tumor staging and prognosis (Figure 1B). Tumor HCETSR levels were lower in cases with poor survival status (vs alive) (Figure 1C), positive lymph node metastasis (vs negative) (Figure 1D), and tumor sizes greater than 5 cm (vs < 5 cm) (Figure 1E). Notably, all HCC patients with positive lymph node metastasis exhibited low expression of HCETSR. Introducing the tumor HCETSR levels into the AUROC to predict the probability of mortality yielded an area under the curve of 0.763 (Figure 1F). The log‐rank test indicated that HCC patients with low tumor HCETSR levels had a significantly lower overall rate of survival compared with those with high levels, with a 3‐year overall survival rate of 42.9% in the low HCETSR group (Figure 1G). Cox analysis identified HCETSR expression as a protective factor of HCC mortality, reducing the risk of disease mortality by 63.3% (HR:0.367, 95% CI: 0.194‐0.696) (Figure 1I).

### 2.3 HCETSR Inhibits the Malignancy of HCC In Vitro and In Vivo

**Figure 2:**
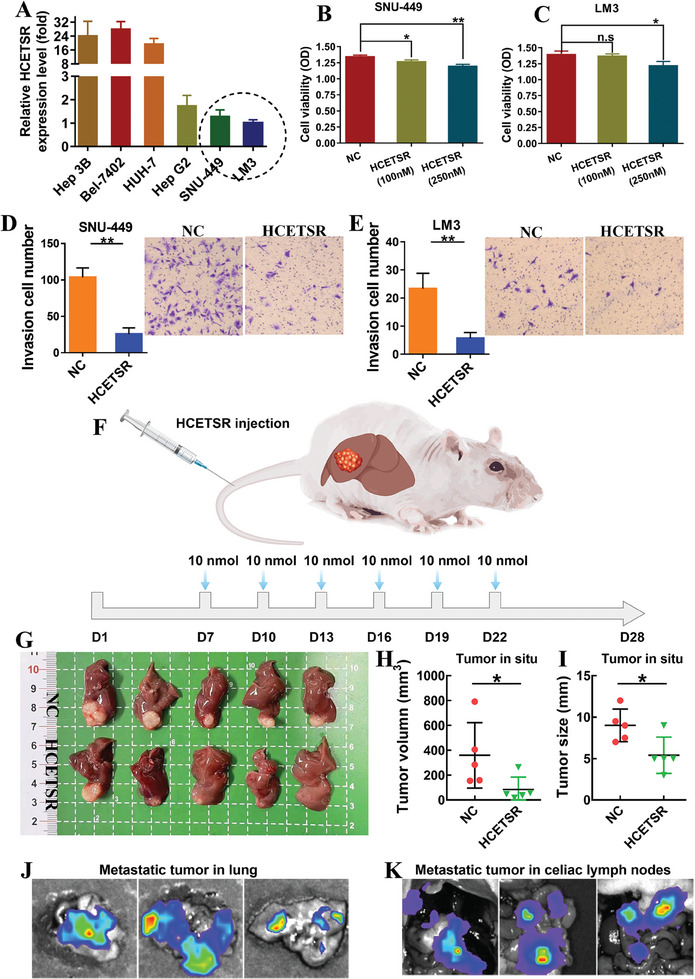
HCETSR inhibits the malignancy of HCC cells in vitro and in vivo. A) The relative expression levels of HCETSR in HCC cell lines are shown. B,C) CCK‐8 assay demonstrates the proliferation of SNU‐449 and LM3 after transfection with HCETSR or its negative control (NC). D,E) Transwell assays with Matrigel show the invasion capabilities of SNU‐449 and LM3 after transfection with HCETSR or its negative control. F) Schematic representation of HCETSR tail vein injection in nude mice. G–I) In vivo imaging of HCC in situ mice models shows liver tumor growth after injection of NC and HCETSR (*n* = 5 per group). Tumor volume and size are measured. J,K) Imaging reveals pulmonary and abdominal metastases in the NC group (3/5). Cell functional assays were performed with three individual experiments. ^*^ *p* < 0.05, ^**^ *p* < 0.01. ns, nonsignificant; NC, negative control.

We first evaluated the function of HCETSR in HCC in vitro by synthesizing and transfecting HECTSR mimics into SNU‐449 and LM3 cells, which had the lowest HCETSR expression levels (**Figure** **2**A). Four days post‐transfection, the optical density significantly decreased in SNU‐449 and LM3 compared to negative control (Figure 2B,C). Additionally, transwell with Matrigel showed a reduction in invasive cell numbers (Figure 2D,E). These results preliminarily suggest that HCETSR inhibits the proliferation and invasion of HCC in vitro.

In vivo, we constructed an HCC in situ nude mouse model to evaluate the effects of HCETSR on HCC (Figure 2F). On D7, successfully established nude mice were selected through fluorescent live imaging and then randomized. HCETSR agomir analog was injected into mice via tail vein every three days. Finally, we found that HECTSR significantly reduced the tumor burden in the liver, both in tumor volume and size (Figure 2G–I), with no distant metastasis observed. In the control group, three out of five (3/5) showed distant metastasis both in lung and abdominal cavity (Figure 2J,K). These results suggest that HCETSR has potential therapeutic value for HCC in vivo.

### 2.4 HCETSR Binds to SPTBN1

**Figure 3:**
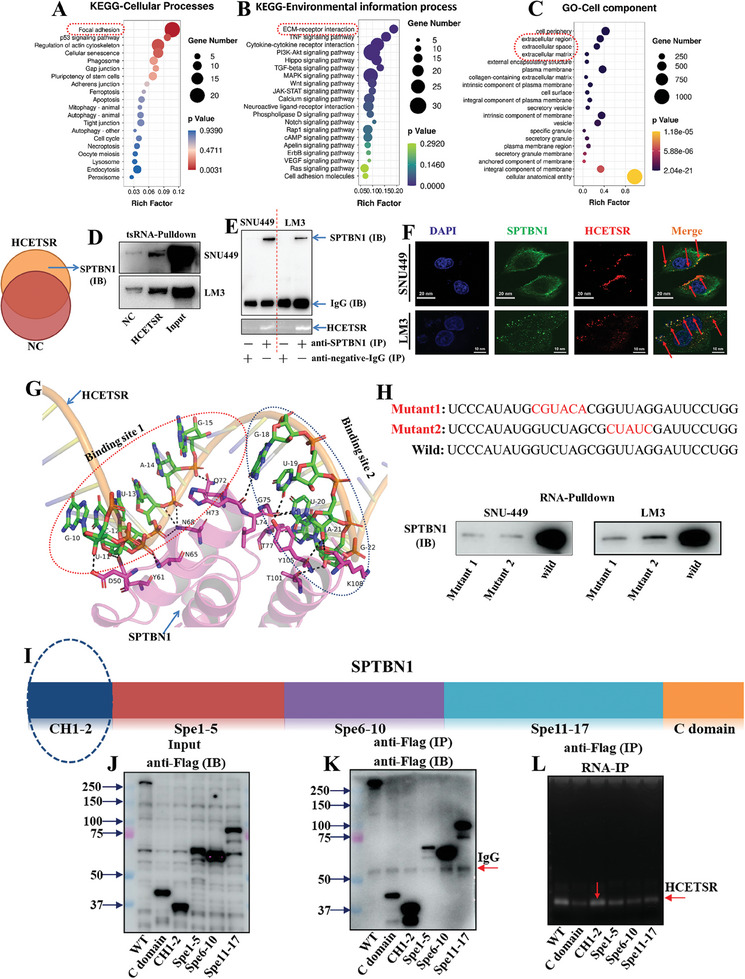
HCETSR can bind with SPTBN1. A–C) KEGG and GO analysis from RNA sequencing data after transfecting SNU‐449 with HCETSR highlight significant pathways and cellular components. D) RNA pulldown assay shows that biotinylated HCETSR captures SPTBN1, detected by immunoblotting with anti‐SPTBN1 antibody. E) RNA immunoprecipitation confirms that anti‐SPTBN1 specifically recognized SPTBN1 can bind with HCETSR. F) Immunofluorescence reveals colocalization of Cy3‐labeled HCETSR and FITC‐labeled endogenous SPTBN1 on the cell membrane (red arrow). G) Schematic diagram depicting molecular docking between SPTBN1 and HCETSR. H) RNA pulldown assay shows binding of two HCETSR mutants and wild‐type HCETSR to SPTBN1. I) Schematic representation of wild‐type SPTBN1 and its five domains. J–L) After transfecting HCC cells with five Flag‐labeled domains of SPTBN1 or wild‐type SPTBN1, expression is confirmed by anti‐Flag antibody (J). The five Flag labeled domains of SPTBN1and wild‐type of SPTBN1 were specifically immunoprecipitated with anti‐Flag antibody (K). RNA immunoprecipitation shows that the CH1‐2 domain of SPTBN1 binds to HCETSR similarly to wild‐type of SPTBN1 (L). KEGG, kyoto encyclopedia of genes and genomes; GO, Gene Ontology; NC, negative control; IB, immunoblotting; IP, immunoprecipitating.

To explore the mechanisms by which HCETSR inhibited the HCC malignancy, we first transfected HCETSR into SNU‐449 cell and performed RNA sequencing (Appendix SC, Supporting Information). KEGG analysis revealed significant enriched pathways in focal adhesion and ECM‐receptor interaction (**Figure** **3**A,B; Appendix SD, Supporting Information). Gene ontology analysis indicated a focus on cell periphery and extracellular region (Figure 3C; Appendix SE, Supporting Information). These results suggest HCETSR may affect intercellular adhesion by regulating cytoskeleton components and signaling transduction.

We then performed biotin‐streptavidin pull‐down assays by transfecting biotinylated HCETSR or negative control into SNU‐449 and LM3. Using LC‐MS/MS of the pull‐down products, we identified SPTBN1, a vital component of the spectrin‐actin cytoskeleton, as a potential HCETSR binding protein (Appendix SF, Supporting Information). Biotin‐streptavidin RNA pull‐down assays with immunoblotting confirmed that HCETSR bind with SPTBN1, in both SNU‐449 and LM3 (Figure 3D). RNA‐IP with PCR confirmed that endogenous SPTBN1 could specifically bind to HCETSR (Figure 3E). Immunofluorescence demonstrated that FITC‐labeled endogenous SPTBN1 focused on the periphery of SNU‐449 and LM3. Cy3‐labeled HCETSR also tended to converge on cell membrane and obviously co‐localized with SPTBN1 (Figure 3F). These results suggest an interaction between SPTBN1 and HCETSR.

Molecular docking showed that two regions of HCETSR (GUCUAG at positions 10–15 and GUUAG at positions 18–22) likely form stem‐loops that bind to SPTBN1 (Figure 3G). Two mutant biotinylated‐HCETSR pull‐down assay with immunoblotting confirmed reduced binding of SPTBN1, compared to wild‐type HCETSR (Figure 3H). We further designed and cloned five continuous Flag‐labeled domains and one wild‐type (See sequence in Appendix SA Table S2, Supporting Information). IP with immunoblotting (anti‐Flag) showed that the corresponding domains and wild‐type could be translated and recognized by the anti‐Flag antibody (Figure 3J,K). RIP with PCR demonstrated that CH1‐2 domain (1‐278 amino acids) was enriched with abundant HCETSR (Figure 3L). These results indicated that CH1‐2 domain of SPTBN1 was the primary site for the loop of HCETSR.

### 2.5 HCETSR Interrupts the Combination of SPTBN1 and Catenin Complex

**Figure 4:**
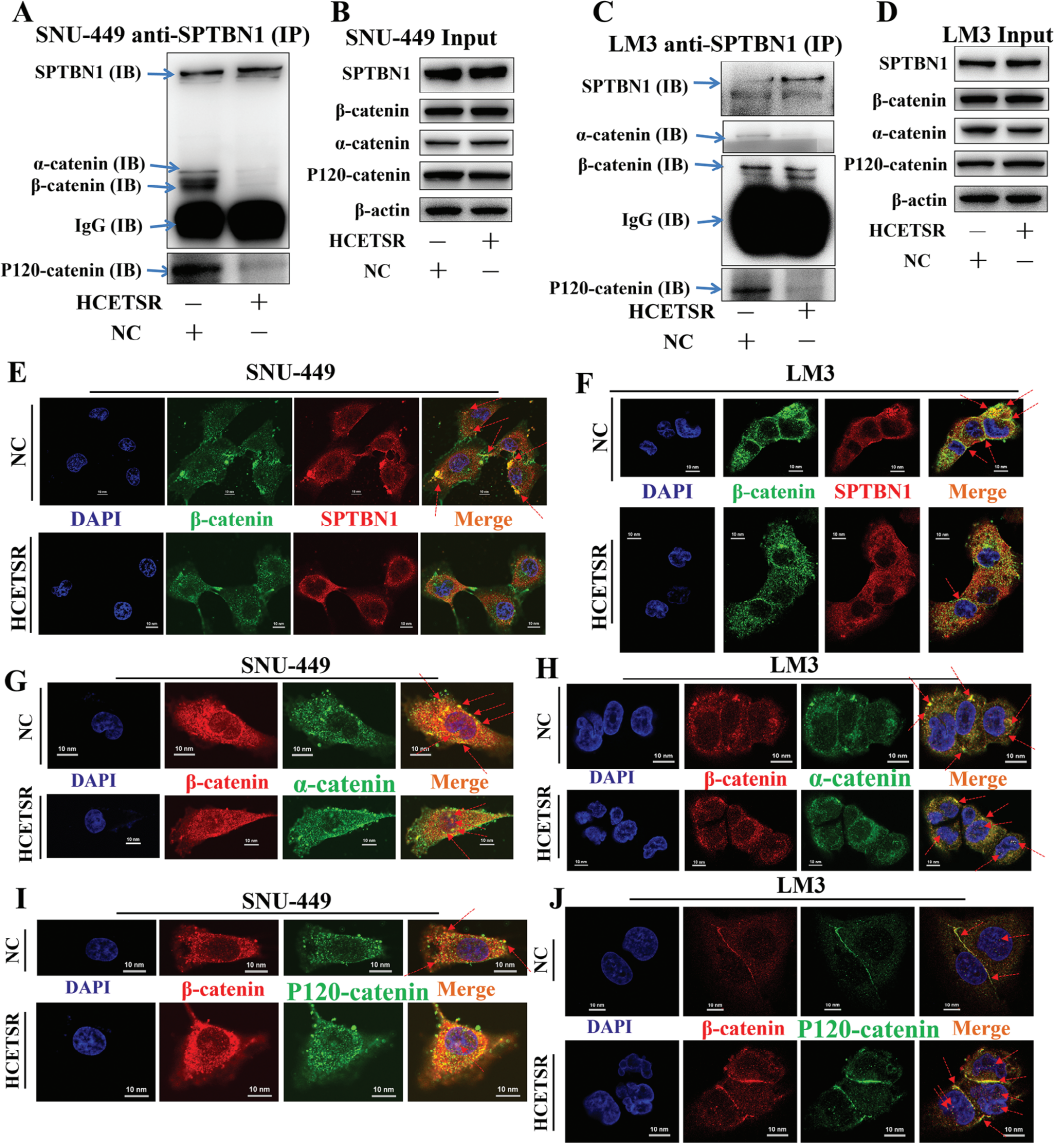
HCETSR interrupts the binding of SPTBN1 and catenin complex. A–D) Co‐immunoprecipitation reveals that SPTBN1 interacts with β‐catenin, α‐catenin, and P120‐catenin. Immunoprecipitation with anti‐SPTBN1 antibody followed by immunoblotting with anti‐β‐catenin antibody, anti‐α‐catenin antibody, anti‐P120‐catenin antibody, and anti‐SPTBN1 antibody. A and C show immunoprecipitation results from SNU‐449 and LM3, where fewer β‐catenin, α‐catenin, and P120‐catenin bind to SPTBN1 post‐HCETSR transfection. B and D show HCETSR do not affect the total expression levels of SPTBN1, β‐catenin, α‐catenin, and P120‐catenin in SNU‐449 and LM3. E,F) Immunofluorescence shows colocalization of β‐catenin and SPTBN1 in SNU‐449 (E) and LM3 (F), with HCETSR reducing the association of SPTBN1 with β‐catenin at the cell membrane. G–J) Immunofluorescence shows colocalization of β‐catenin, α‐catenin, and P120‐catenin, components of the catenin complex, in cell membrane and nuclei. HCETSR promotes their nuclear translocation. G and H show β‐catenin and α‐catenin in SNU‐449 and LM3, respectively. I and J show β‐catenin and P120‐catenin in SNU‐449 and LM3. NC, negative control; IB, immunoblotting; IP, immunoprecipitating.

We further explored the effects following the binding of HCETSR and SPTBN1. We hypothesized that SPTBN1, β‐catenin, α‐catenin, and P120‐catenin may interact with each other as components of cytoskeleton. IP (anti‐SPTBN1) with immunoblotting in the negative control showed that β‐catenin, α‐catenin, and P120‐catenin formed a complex, and SPTBN1 interacted with this catenin complex. While HCETSR significantly disrupted the interaction between SPTBN1 and the catenin complex (**Figure** **4**A–D). HCETSR pull‐down with LC‐MS/MS also failed to detect the catenin complex (Appendix SF, Supporting Information).

Immunofluorescence showed that the colocalization of SPTBN1 and β‐catenin at the cell membrane was disrupted by HCETSR (Figure 4E,F). The β‐catenin, α‐catenin, and P120‐catenin formed a catenin complex distributed around the cell membrane (Figure 4G–J). HCETSR also caused increased translocation of β‐catenin into the nucleus.

### 2.6 HCETSR Reduces Proteasomal Degradation of β‐catenin and Synthesis of Nascent β‐catenin

**Figure 5:**
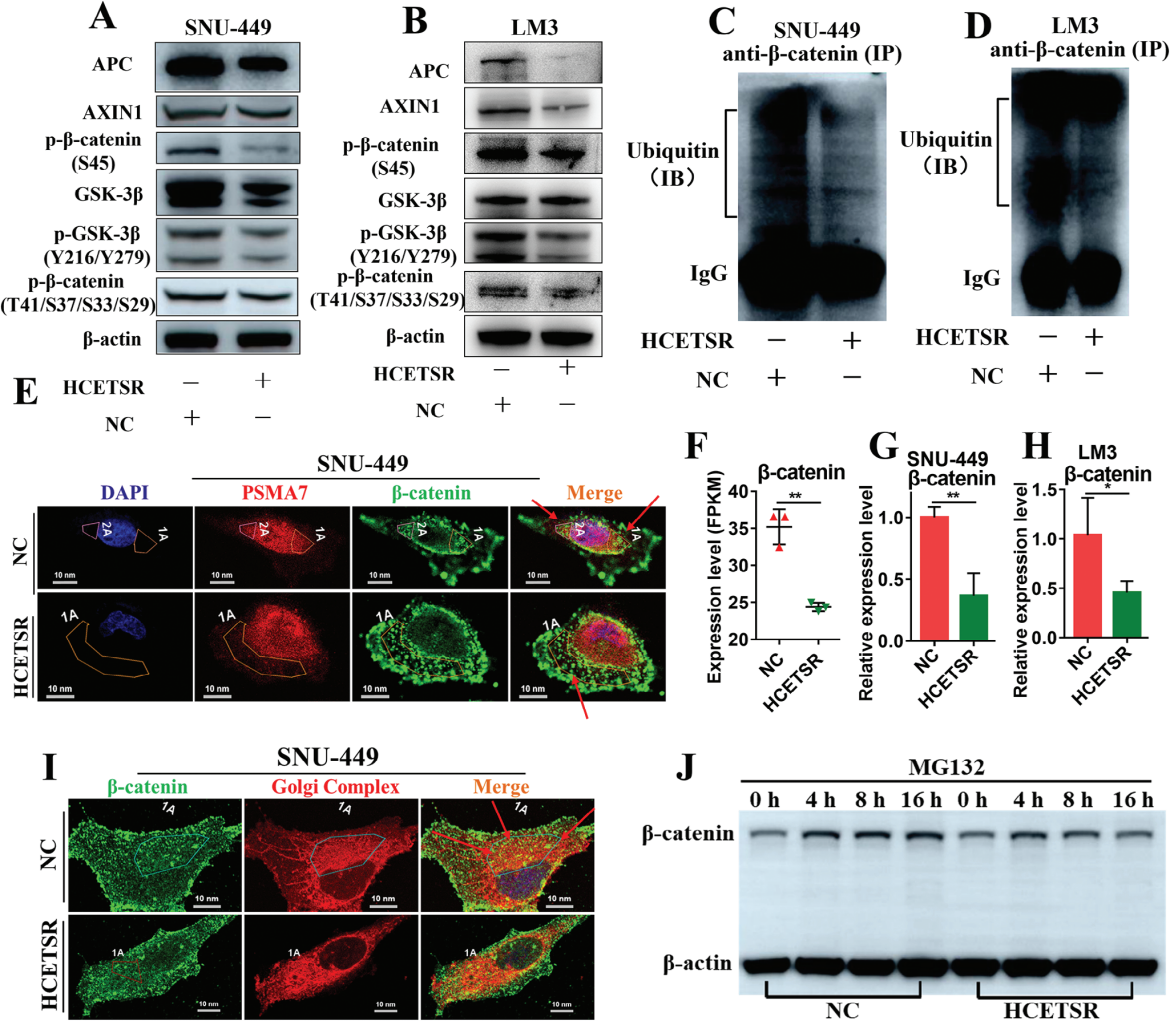
HCETSR inhibits β‐catenin proteasomal degradation and nascent β‐catenin synthesis. A,B) Western blot analysis shows the expression of APC, AXIN1, phosphorylated‐β‐catenin (S45), GSK‐3β, phosphorylated‐GSK‐3β/GSK‐3α (Y216/Y279), phosphorylated‐β‐catenin (T41/S37/S33/S29) in SNU‐449 (A) and LM3 (B) after HCETSR transfection. C,D) Immunoprecipitation shows the ubiquitin level of β‐catenin in SNU‐449 (C) and LM3 (D) post‐HCETSR transfection. Immunoprecipitated with anti‐β‐catenin antibody and immunoblotted with anti‐ubiquitin antibody. E) HCETSR inhibits the colocalization of PSMA7 and β‐catenin in SNU‐449, detected by immunofluorescence. HCETSR reduces the intake of β‐catenin into proteasome. F–H) The transcription level of β‐catenin is detected by RNA sequencing (F), and by qRT‐PCR in SNU‐449 (G), and LM3 (H). I) HCETSR inhibits the colocalization of Golgi complex and β‐catenin in SNU‐449. J) Western blot analysis under proteasomal degradation inhibition by MG132 shows that HCETSR decreases nascent β‐catenin expression. RT‐qPCR data are presented as mean ± SD from three individual experiments. ^*^ *p* < 0.05 and ^**^ *p* < 0.01.

We first analyzed the influence of HCETSR on the Wnt‐Off pathway, focusing on β‐catenin, a pivotal protein in the Wnt signaling cascade. HCETSR reduced the expression levels of APC and Axin1, subsequently inhibiting the phosphorylation of β‐catenin at S45. This reduction led to decreased GSK3β phosphorylation at Y279, further inhibiting the phosphorylation of β‐catenin at T41/S37/S33/S29. Ultimately, these molecular events culminated in the inhibition of β‐catenin ubiquitination (**Figure** **5**A,B). IP with immunoblotting confirmed that HCETSR inhibited the level of β‐catenin ubiquitination (Figure 5C,D). PSMA7, a key subunit of the 20S proteasome,^[^ ^41^ ^]^ was used to represent proteasome in cell. Under the influence of HCETSR, colocalization of β‐catenin and PSMA7 were significantly reduced, reflecting that the loss of β‐catenin was intake by proteasomal degradation (Figure 5E).

Although HCETSR reduced the degradation of β‐catenin, β‐catenin protein level did not increase (Figure 4B–D), suggesting inhibited synthesis of nascent β‐catenin. RNA sequencing and qRT‐PCR confirmed that HCETSR reduced β‐catenin transcription levels (Figure 5F–H). Immunofluorescence showed that HCETSR diminished the neosynthesis of β‐catenin within the Golgi complex (Figure 5I). Meanwhile, under the influence of proteasomal inhibition MG132, HCETSR significantly reduced the expression of nascent β‐catenin (Figure 5J). These findings suggest that HCETSR inhibits the synthesis of nascent β‐catenin.

### 2.7 HCETSR Inhibits the LEF1 Activity

**Figure 6:**
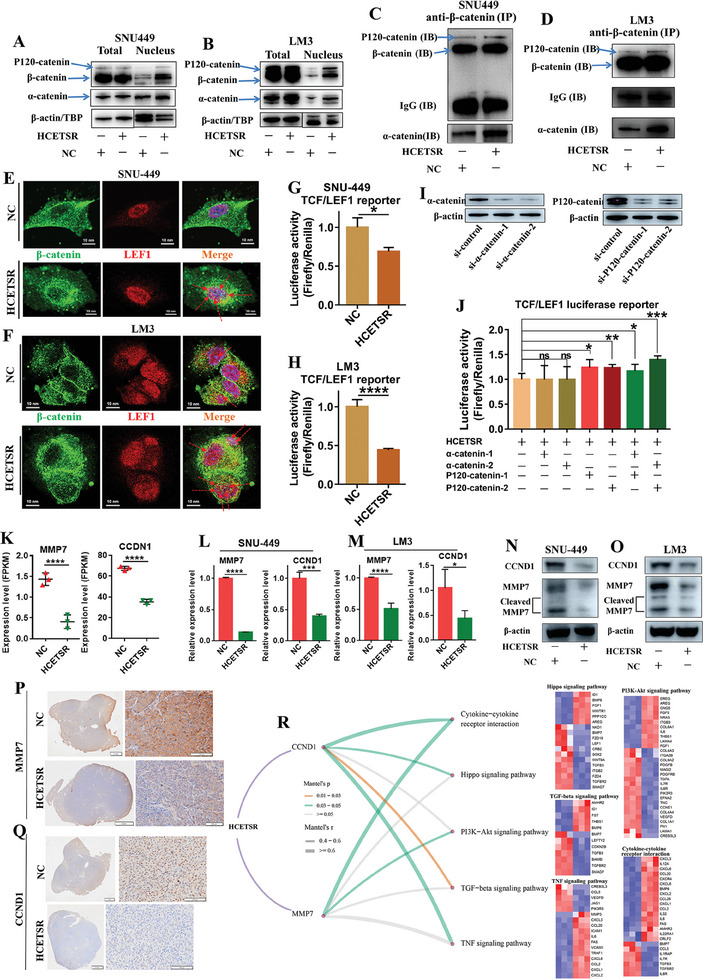
HCETSR regulates the nuclear translocation of catenin complex and inhibits LEF1 transcription factor activity. A,B) Western blot analysis shows that HCETSR increases the nuclear expression of β‐catenin, α‐catenin, and P120‐catenin in SNU‐449 (A) and LM3 (B). C,D) Immunoprecipitation indicates that HCETSR enhances the binding of α‐catenin, and P120‐catenin with β‐catenin in SNU‐449 (C) and LM3 (D). Immunoprecipitation was performed with anti‐β‐catenin antibody, and immunoblotting was done with anti‐α‐catenin antibody, anti‐P120‐catenin antibody, and anti‐β‐catenin antibodies. E,F) Immunofluorescence shows colocalization of β‐catenin and LEF1 after transfected with HCETSR in SNU‐449 (E) and LM3 (F) (red arrow). G,H) Dual‐luciferase reporter assay shows that HCETSR reduces the luciferase activity of LEF1 transcription factor in SNU‐449 (G) and LM3 (H). I) Western blot analysis shows siRNA‐mediated knockdown of α‐catenin and P120‐catenin. J) Dual‐luciferase reporter assay indicates the effect of P120‐catenin and α‐catenin knockdown on LEF1 transcription factor luciferase activity after being transfected with HCETSR. K–M) RNA sequencing (K) and qRT‐PCR data from SNU‐449 (L) and LM3 (M) show that HCETSR reduces the transcription levels of MMP7 and CCND1. N,O) Western blot analysis shows that HCETSR inhibits the expression of MMP7 and CCND1 in SNU‐449 and LM3. P,Q) Immunohistochemistry demonstrates that HCETSR inhibits the expression of MMP7 and CCND1 in the HCC in situ mouse model. R) RNA sequencing and Mantel's test analysis display correlations between MMP7 and CCND1 with significant pathways influenced by HCETSR. The color represents the P value, and the thickness represents the correlation. Five heatmaps show differentially expressed genes in five pathways, influenced by HCETSR. Luciferase reporter assay and RT‐qPCR data are presented as mean ± SD of three individual experiments. ^*^ *p* < 0.05, ^**^ *p* < 0.01, ^***^ *p* < 0.001, and ^****^ *p* < 0.0001. ns, nonsignificant; NC, negative control; IB, immunoblotting; IP, immunoprecipitating.

Immunofluorescence revealed that HCETSR increased the nuclear translocation of β‐catenin, α‐catenin, and P120‐catenin (Figure 4G–J). After nuclear protein separation, it was observed that HCETSR facilitated the accumulation of the catenin complex into the nucleus (**Figure** **6**A,B). IP with immunoblotting showed increased binding of β‐catenin with α‐catenin and P120‐catenin (Figure 6C,D). Thus, HCETSR promotes the nuclear translocation of the catenin complex.

Following the nuclear translocation of β‐catenin facilitated by HCETSR, the binding between β‐catenin and LEF1 also increased (Figure 6E,F). Interestingly, dual luciferase reporter assays showed that HCETSR inhibited the activity of the LEF1 transcription factor, potentially due to the effects of α‐catenin and P120‐catenin (Figure 6G,H). By synthesizing siRNAs targeting α‐catenin and p120‐catenin separately, we successfully downregulated their expression levels (Figure 6I). Co‐transfection of siRNAs with HCETSR confirmed that the decrease in P120‐catenin expression, rather than α‐catenin, alleviated the inhibitory effect of HCETSR on LEF1 transcription factor (Figure 6J).

MMP7 and CCDN1, downstream effectors of LEF1, are crucial factors involved in tumor metastasis and cell cycle progression.^[^ ^42^, ^43^, ^44^ ^]^ RNA sequencing and qRT‐PCR demonstrated that HCETSR inhibition of LEF1 led to suppression of MMP7 and CCDN1 transcription (Figure 6K–M). Western blot and immunohistochemistry analysis of animal specimens confirmed reduced protein levels of MMP7 and CCDN1 due to HCETSR inhibition (Figure 6N–Q). RNA sequencing analysis with Mantel's test revealed correlations between HCETSR‐mediated suppression of MMP7 and CCDN1 and HCETSR‐mediated alterations in top five core cancer‐related pathways (Figure 6R). Specifically, CCND1 regulated the Hippo signaling pathway, cytokine‐cytokine receptor interaction, the TGF‐beta signaling pathway, and the TNF signaling pathway, while MMP7 influenced cytokine‐cytokine receptor interaction and the PI3K‐Akt signaling pathway. Thus, HCETSR modulates these five cores signaling pathways by inhibiting two oncogenic factors, CCDN1 and MMP7, ultimately suppressing HCC malignance (Figure 6R).

### 2.8 HCETSR is Spliced from tRNA‐Glu/TTC

**Figure 7:**
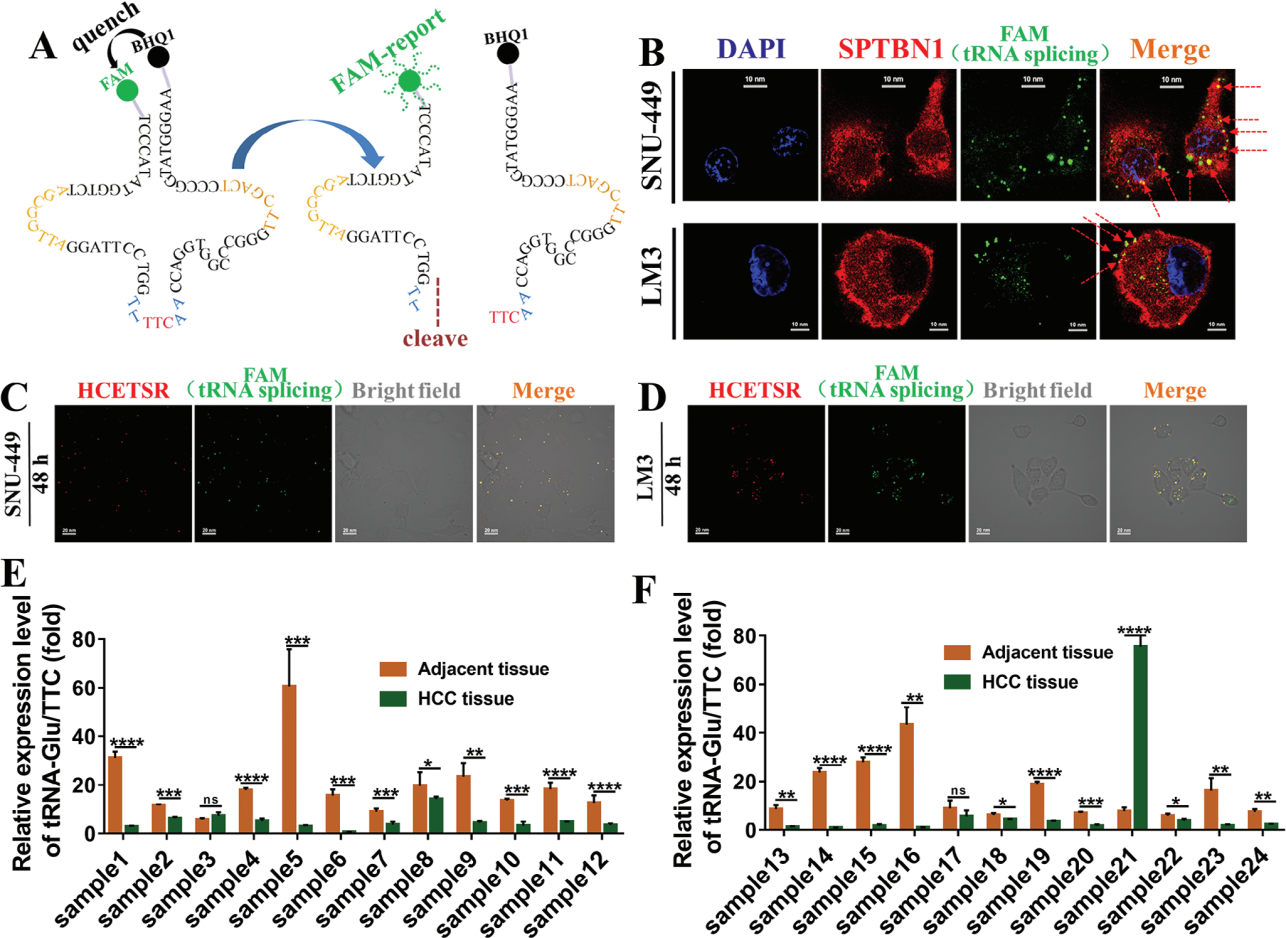
HCETSR is generated from tRNA‐Glu/TTC. A) A schematic diagram illustrates the process of tRNA‐Glu/TTC cleavage into HCETSR, with tRNA‐Glu/TTC labeled with reporter dye FAM at its 5′ end and quencher dye BHQ1 at its 3′ end. Detection of significant FAM signal indicates tRNA‐Glu cleavage. B) Immunofluorescence shows colocalization of SPTBN1 and FAM signal after transfected with FAM/BHQ1 labeled tRNA‐Glu for 72 h. C,D) Immunofluorescence detects colocalization of FAM and Cy3 signal in SNU‐449 and LM3 co‐transfected with FAM/BHQ1 labeled tRNA‐Glu and Cy3 labeled HCETSR. E,F) qRT‐PCR shows downregulation of tRNA‐Glu in HCC tissues compared to adjacent non‐tumor tissues (24 pairs). RT‐qPCR data are presented as mean ± SD from three individual experiments. ^*^ *p* < 0.05, ^**^ *p* < 0.01, ^***^ *p* < 0.001, and ^****^ *p* < 0.0001. ns, nonsignificant;.

First, we evaluated whether HCETSR is generated through the cleavage of tRNA‐Glu/TTC. Synthetical tRNA‐Glu/TTC was fluorescently labeled with reporter dye FAM on its 5′ end and quencher dye BHQ1 on its 3′ end. Cleavage of tRNA‐Glu relieved the quenching effect of BHQ1 on FAM, enabling the observation of the FAM signal (**Figure** **7**A). Immunofluorescence indicated that the FAM signal primarily localized to the cell membrane and co‐localized with SPTBN1 (Figure 7B), consistent with previous results in Figure 3F (Cy3‐labeled HCETSR). Furthermore, co‐transfection of tRNA‐Glu/TTC and cy3‐labeled HCETSR into HCC cells demonstrated nearly‐complete co‐localization of HCETSE with the FAM signal (Figure 7C,D). These results indicate that the product of tRNA‐Glu/TTC cleavage functions identically to HCETSR. Thus, HCETSR originates from the cleavage of tRNA‐Glu/TTC.

We further investigated the expression levels of tRNA‐Glu/TTC in HCC. Using 24 pairs of HCC tissues and adjacent normal tissues, we found that 21 pairs exhibited significantly lower expression of tRNA‐Glu/TTC in HCC (Figure 7E,F), consistent with the findings for HCETSR in Figure 1A. This significant downregulation of tRNA‐Glu/TTC in HCC was directly linked to the low expression of HCETSR.

### 2.9 Regulation of HCETSR and tRNA‐Glu/TTC by Spliceosome and Dicer1

**Figure 8:**
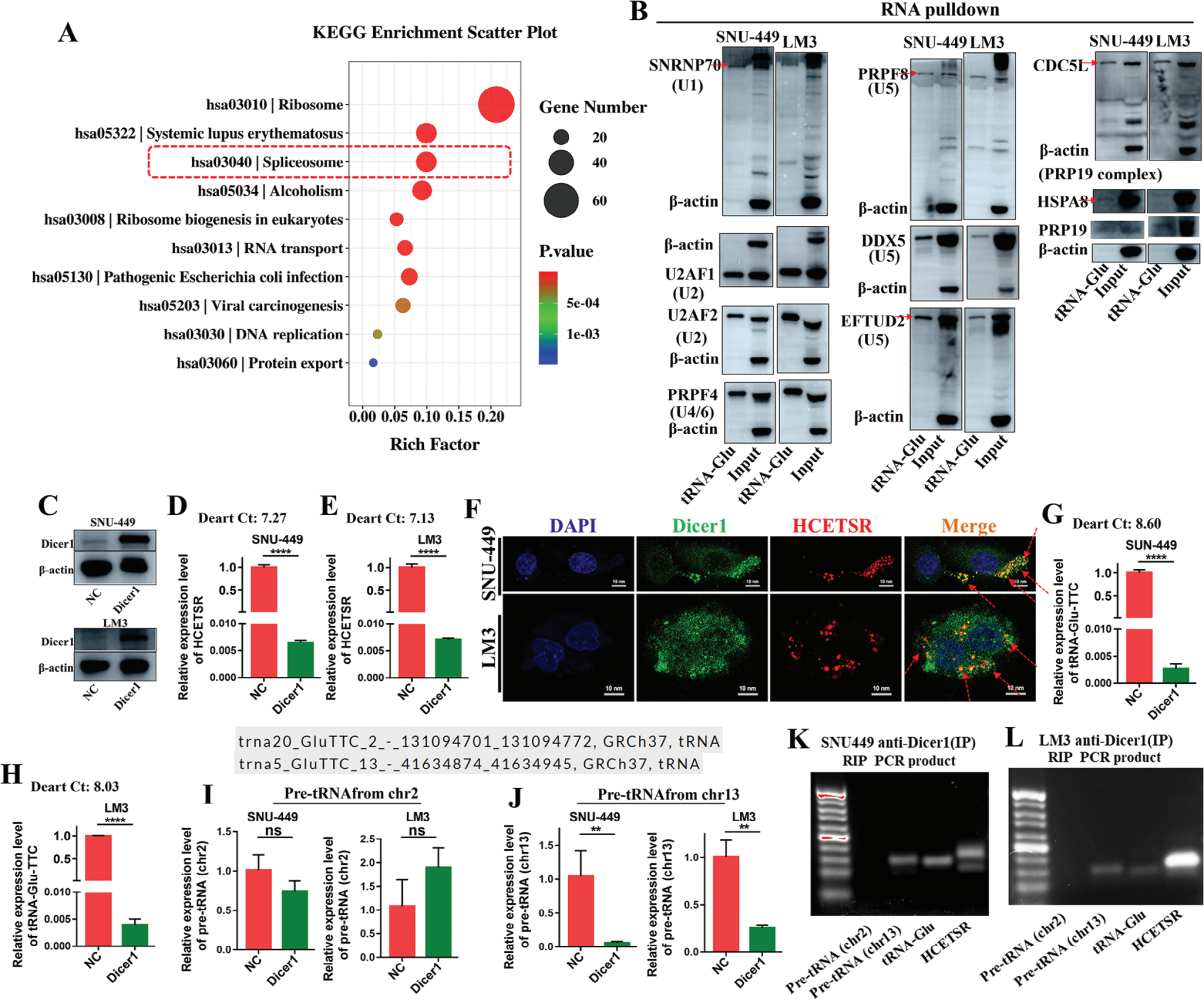
Spliceosome and Dicer1 regulate the generation of tRNA‐Glu/TTC and HCETSR. A) KEGG analysis based on proteins identified by tRNA pulldown and mass spectrometry. B) tRNA pulldown with immunoblotting detects spliceosome components binding to tRNA‐Glu, including SNRNP70, U2AF1, U2AF2, PRPF4, PRPF8, DDX5, EFTUD2, CDC5L, HSPA8, and PRP19. C) Western blot analysis shows Dicer1 overexpression in SNU‐449 and LM3 cell after transfection with Dicer1 plasmid. D,E) qRT‐PCR shows decreased HCETSR expression in SNU‐449 (D) and LM3 (E) cells post‐Dicer1 overexpression. F) Immunofluorescence detects colocalization of Dicer1 and Cy3 labeled HCETSR in SNU‐449 and LM3. G,H) qRT‐PCR shows that Dicer1 overexpression decreases the expression of tRNA‐Glu in SNU‐449 (G) and LM3 (H). I,J) qRT‐PCR shows the expression of pre‐tRNA‐Glu from chr2 and chr9 in SNU‐449 and LM3 after Dicer1 transfection. K,L) RNA immunoprecipitation shows Dicer1‐bound RNA in SNU‐449 (K) and LM3 (L), with PCR detection of chr2‐pre‐tRNA, chr9‐pre‐tRNA, tRNA‐Glu/TTC, and HCETSR. Immunoprecipitation was performed with anti‐Dicer1 antibody. RT‐qPCR data are presented as mean ± SD from three individual experiments. ^**^ *p* < 0.01 and ^****^ *p* < 0.0001. KEGG, kyoto encyclopedia of genes and genomes; ns, nonsignificant; IP, immunoprecipitating.

Hence, the underlying regulation mechanisms of tRNA‐Glu/TTC or HCETSR warrant further exploration. We performed biotin‐streptavidin RNA pull‐down assays by transfecting biotinylated tRNA‐Glu/TTC (Appendix SG, Supporting Information). KEGG analysis from LC‐MS/MS of the products suggested that tRNA‐Glu/TTC may be cleaved by the spliceosome (**Figure** **8**A; Appendix SH, Supporting Information). RNA pull‐down with immunoblotting showed that tRNA‐Glu/TTC binds to numerous spliceosomal components, including small nuclear ribonucleoproteins (snRNPs) such as SNRNP70 (U1), U2AF1 (U2), U2AF2 (U2), PRPF4 (U4/6), PRPF8 (U5), DDX5 (U5), EFTUD2 (U5), and the PRP19 complex, which includes CDC5L, HSPA8, and PRP19(Figure 8B). Thus, the spliceosome promotes the regulation of tRNA‐Glu/TTC in HCC.

Specifically, Dicer1 is involved in the splicing and generation of noncoding RNA molecules.^[^ ^45^ ^]^ After overexpressing Dicer1 in HCC cell lines (Figure 8C), we observed a significant reduction in HCETSR level (Figure 8D,E). Immunofluorescence revealed colocalization between Dicer1 and HCETSR, implicating Dicer1 in the cleavage of HCETSR (Figure 8F). It prompts further investigation into whether Dicer1 is involved in the genesis process of HCETSR. Dicer1 overexpression also suppressed the transcriptional levels of tRNA‐Glu/TTC (Figure 8G,H). Further upstream tracing to precursor tRNA‐Glu (pre‐tRNA‐Glu) revealed its potential transcriptional origins from chromosomes 2 and 9 (chr2 and 9). Dicer1 overexpression specifically affected pre‐tRNA‐Glu transcription from chr9 while leaving chr2‐derived transcription unaffected (Figure 8I,J). RNA immunoprecipitation confirmed that Dicer1‐bound RNAs included chr9‐pre‐tRNA, tRNA‐Glu/TTC, and HCETSR, but did not include chr2‐pre‐tRNA (Figure 8K,L). These findings demonstrate Dicer1's involvement in the genesis of HCETSR.

### 2.10 Wnt Pathway Does Not Affect the Expression of HCETSR

The mechanism of HCETSR intersects to some extent with the canonical Wnt‐on and Wnt‐off pathways. We examined whether the Wnt pathway regulates HCETSR expression. Two Wnt pathway activators, Wnt3a and Dalosirvat, and two inhibitors, Salinmycin and Kallistatin were used. Although Wnt3a and Dalosirvat activated the Wnt pathway and Salinomycin and Kallistatin inhibited it, none of these agents significantly affected HCETSR expression (Appendix SA Figure S2, Supporting Information).

## 3 Discussion

tsRNAs, recognized as a rising star, are gaining attention for their role in tumor regulation. tsRNAs can be classified into several groups: 5′‐tRNA halves (tiRNA‐5), 3′‐tRNA halves (tiRNA‐3), itRF, tRF‐5, and tRF‐3. tiRNAs are specifically cleaved in the anticodon loop of mature tRNAs.^[^ ^46^ ^]^ They repress protein translation, functioning similarly to miRNAs.^[^ ^47^ ^]^ Studies have shown that tiRNA‐5 can inhibit FZD3 and PIK3CD, regulating tumor progression.^[^ ^48^, ^49^ ^]^ Additionally, tiRNA such as tiRNA‐Val‐CAC‐2 can interact with FUBP1 to promote pancreatic cancer metastasis.^[^ ^50^ ^]^ tRF‐5 and tRF‐3 are derived from the 5′ and 3′ end of mature tRNA, respectively. According to Sarais F et al., HCETSR should be classified as tRF‐5c.^[^ ^51^ ^]^ The regulatory mechanisms of tRF‐5 in tumors are complex and involve interactions with key factors. For instance, AS‐tDR‐007333 binds to HSPB1 to promote NSCLC malignancy,^[^ ^52^ ^]^ and 5′‐tRFCys promotes metastatic and metabolic effects in breast cancer by binding Mthfd1l and Pafah1b1 transcripts.^[^ ^53^ ^]^ In this study, RNA sequencing and LC‐MS/MS analysis indicated that HCETSR controls focal adhesion, ECM‐receptor interaction, and cytoskeleton regulation. The cytoskeleton protein SPTBN1 was identified as a crucial binding partner of HCETSR.

SPBTN1, first discovered in erythroid cells, plays a critical role in the mechanical stability and elasticity of red blood cells.^[^ ^33^, ^54^ ^]^ SPTBN1 has been implicated in tumor regulation.^[^ ^38^ ^]^ However, its impact on tumors through interaction with other cellular components remains unclear due to its role as a component of the cellular cytoskeleton. Our results show ed that SPTBN1 can bind with the catenin complex, which is interrupted by HCETSR. This disruption leads to changes in the catenin complex signaling, as components of the cellular cytoskeleton dynamically reorganize in cancer cells. Signal transduction can occur when structure changes in the cellular cytoskeleton are accompanied by the movement of its components in cancer cells, even if the levels of these components remain unchanged.

We propose a new concept involving the catenin complex (β‐catenin, P120‐catenin, and α‐catenin) which can detach from the cytoskeleton and translocate to the nucleus. α‐catenin and β‐catenin have been reported to co‐localize in the nucleus, which is associated with a reduction in LEF1‐dependent transcriptional activity.^[^ ^55^ ^]^ The expression of α‐catenin prevents the nuclear translocation of β‐catenin induced by EGFR activation in human glioblastoma cells.^[^ ^56^ ^]^ The association of P120‐catenin with Kaiso alleviates sequence‐specific gene repression by dissociating Kaiso from DNA.^[^ ^57^ ^]^ Our study demonstrated that HCETSR disrupts the binding of the catenin complex and SPTBN1, allowing the catenin complex to shuttle from the cell membrane to the nucleus and further inhibiting LEF1 transcriptional activity. Specifically, the effects produced by the nuclear translocation of the catenin complex are mainly due to the inhibitory action of P120‐catenin, rather than α‐catenin.

tRFs, which are products of pre‐tRNA or mature tRNA, are generated under certain circumstances. In the nucleus, pre‐tRNAs are transcribed from tRNA genes by RNA polymerase III, and mature tRNAs are produced by the splicing and modification of pre‐tRNAs.^[^ ^58^ ^]^ tsRNAs can also be produced by endonucleases such as ANG and Dicer, which cleave specific sites on pre‐tRNAs or mature tRNAs.^[^ ^59^, ^60^ ^]^ The production of tRF‐5 series has been reported to be Dicer‐dependent.^[^ ^61^ ^]^ The spliceosome, which catalyzes splicing, consists of the spliceosomal small nuclear ribonucleoproteins (U1, U2, and U4/U6·U5 snRNPs) as well as additional non‐consensus motifs provided by splicing and coding domains.^[^ ^62^ ^]^ In this study, we confirmed that the upstream region of HCETSR is tRNA‐Glu/TCC. The cleavage product of tRNA‐Glu/TCC exhibits the same function as synthetically produced HCETSR. tRNA‐Glu/TCC is significantly downregulated in HCC, directly causing the low expression of HCETSR. Both Dicer1 and the spliceosome are involved in the cleavage process of HCETSR.

We reviewed the Canonical Wnt‐on and Wnt‐off pathways. The Wnt‐off pathway promotes the degradation of β‐catenin in the proteasome. Under the action of APC and AXIN, β‐catenin is first phosphorylated at S45 by Ck1, followed by phosphorylation at T41, S37, S33, and S29 by GSK3β.^[^ ^63^, ^64^ ^]^ This sequence eventually leads to the creation of a β‐Trcp site on the E3 ubiquitin ligase, resulting in β‐catenin ubiquitin.^[^ ^63^ ^]^ The Wnt‐on pathway facilitates the shuttling of β‐catenin from the membrane to the nucleus. Wnt protein binds to the Frizzled family receptors and LRP5/6,^[^ ^65^ ^]^ recruits Dvl, inhibits APC and AXIN1, and promotes the phosphorylation of LRP5/6 at PPPSP motifs T1479.^[^ ^66^, ^67^, ^68^ ^]^ Our results indicated that HCETSR decreases β‐catenin ubiquitin and proteasomal degradation, aligning with Wnt‐off pathway. HCETSR also stimulates the nuclear translocation of the catenin complex, which differs from the Wnt‐on pathway. Additionally, our study found that two Wnt pathway activators, Wnt3a and Dalosirvat, and two Wnt pathway inhibitors, Salinmycin and Kallistatin, do not affect the expression of HCETSR. Therefore, HCETSR does not interact with the canonical Wnt‐on pathway.

We could not observe the influence of HCETSR deletion on SPTBN1/catenin complex axis. After designing the gRNA and performing Crisper‐Cas9 to knock out HCETSR, extensive cell death occurred, and the remaining colonies failed to be identified. HCETSR knock‐out is lethal for cells due to the deletion of tRNA‐Glu/TTC, which is necessary for cell survival (Appendix SA Figure S3, Supporting Information).

**Figure 9:**
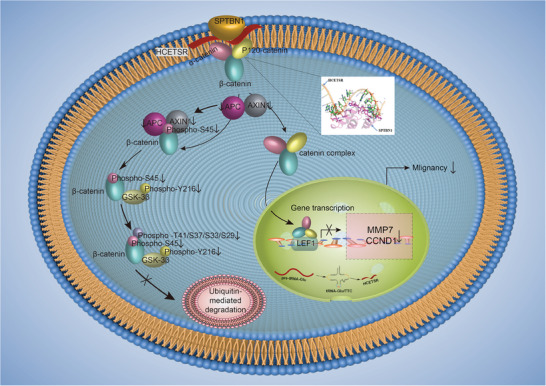
Graphical summary of mechanisms of HCETSR in HCC.

In conclusion, our results shed light on the investigation of tRF in HCC and provide evidence for the role of HCRTSR in inhibiting HCC malignancy (summarized in **Figure** **9**). To the best of our knowledge, this is the first study to demonstrate the novel SPTBN1/catenin complex axis and its biological mechanism from cell membrane and cytoplasm to nucleus, regulated by HCETSR. The splicing of HCETSR and tRNA‐Glu/TTC requires the involvement of the spliceosome and Dicer1, offering further insights into the generation of tRNA and tRF. This finding is expected to provide a novel therapeutic strategy and prognostic prediction for HCC.

## 4 Experimental Section

### tsRNA Sequencing

Small RNAs (sRNAs) were extracted from four HCC samples and adjacent liver tissues. Following quantification and quality control (Agilent 2100 Bioanalyzer, USA), 3′ and 5′ adapters were ligated to the sRNAs. The sRNAs were then amplified via polymerase chain reaction (PCR). The amplified products were gel‐purified, filtered by length, and sequenced using the Illumina HiSeqTM 2500 (Illumina, USA). The resulting libraries were mapped using MINTbase v2.0^[^ ^69^ ^]^ (https://cm.jefferson.edu/mintbase‐v2‐0) to obtain tsRNA profiles. Differentially expressed tsRNAs between HCC samples and adjacent liver tissues were screened using the DESeq R package, with thresholds set at a log_2_ fold change > 1 and adjusted *p* value < 0.05.

### RNA Sequencing

Libraries were prepared according to the instructions of the mRNA‐seq Lib Prep Kit (RK20349, ABclonal, China). Total RNAs were extracted from HCETSR‐treated SNU‐449 and negative control SNU449 cells. mRNAs were enriched using magnetic beads with Oligo dT. Subsequently, complementary DNAs (cDNAs) were synthesized using mRNA as a template, followed by purification with AMPure XP (A63881, Beckman Coulter, USA). The cDNAs underwent PCR amplification for library construction and sequencing using the NovaSeq 6000 system (Illumina, USA). Libraries were mapped to Homo_sapiens_Ensembl_104 (http://may2021.archive.ensembl.org/Homo_sapiens/Info/Index) using the HISAT2 software.^[^ ^70^ ^]^ Differential gene expression analysis was conducted using the DESeq R package.

### Liquid Chromatography with Tandem Mass Spectrometry (LC‐MS/MS)

The protein gel bands were subjected to bleaching, dehydration, and enzymatical digestion. The resulting peptides were desalted. Peptides were separated using the Nano‐HPLC liquid phase system EASY‐nLC1200 (Thermo Scientific, USA). The sample was first adsorbed to a Trap column (100 µm × 20 mm, Thermo Scientific, USA), and then separated using an Analysis column (75 µm × 150 mm, RP‐C18, Thermo Scientific, USA), at a flow rate of 300 nL min^−1^. Peptides were then analyzed by the Q‐Exactive Plus mass spectrometer (Thermo Scientific, USA). Raw mass spectrometer data were mapped to the uniprot‐Human using ProteomeDiscover 2.4.

### Western Blot Analysis

The quantitative protein samples were boiled, electrophoresed on SurePAGE gel (GenScript, China), and transferred onto equilibrated polyvinylidene difluoride membranes (Thermo Scientific, USA). The membranes were blocked with 5% non‐fat milk powder for 1 h at room temperature, incubated with primary antibodies at 4 °C overnight, followed by secondary antibodies for 1 h at room temperature. Detection was performed using an enhanced chemiluminescence (ECL) system (Biotanon, China) with FDbio‐FemtoECL (FD8380, Fudebio, China). The 38 antibodies used in study are shown in Appendix SA Table S1 (Supporting Information).

### Co‐Immunoprecipitation (CO‐IP)

Protein A/G magnetic beads (HY‐K0202, MCE, USA) were pre‐treated with 0.1% Tween‐20 (HY‐141415, MCE, USA) and incubated with the primary antibody at 4 °C for 2 h. Lysis buffer contained 20 mm Tris‐HCl (FD8878, Fudebio, China), 100 mm KCl (P3911, Sigma–Aldrich, USA), 5 mm MgCl_2_ (M8266, Sigma–Aldrich, USA), 0.5% NP‐40 (FD7949, Fudebio, China), Protease inhibitor (FD1001, Fudebio, China), 40 U mL^−1^ Rnase inhibitor (M0314, New England Biolabs, China), and DEPC‐water (FD3620, Fudebio, China), PH 7.5. After the prepared HCC cells were lysed with lysis buffer, cell lysates were incubated with antibody‐conjugated beads overnight at 4 °C. Beads were washed and rinsed 3 times with pre‐cooled lysis buffer, boiled in lysis buffer and SDS loading buffer (FD002, Fudebio, China) for 10 min. The immunoprecipitated proteins were analyzed by western blotting.

### RNA Immunoprecipitation (RIP)

RIP was performed to validate RNA‐protein interactions. The cell preparation, protein A/G magnetic beads pretreatment, cell lysis, and immunoprecipitation steps followed the same protocol as for Co‐IP. After capturing the protein‐RNA complex with antibody‐conjugated beads, RNAs were extracted using the RNA‐Quick purification kit (RN001, esunbio, China) for cDNA synthesis.

### Biotinylated RNA Pulldown

HCC cells were transfected with biotinylated HCETSR or tRNA‐Glu/TTC (SunYa, China) for 48 h and lysed with lysis buffer (20 mm Tris‐HCl, 100 mm KCl, 5 mm MgCl_2_, 0.5% NP‐40, Protease inhibitor, 40 U mL^−1^ Rnase inhibitor, and DEPC‐water, PH 7.5). Streptavidin Magnetic Beads (HY‐K0208, MCE, USA) were pretreated with 10 mm Tris‐HCl, 1 mm EDTA (HY‐Y0682, MCE, USA), 1 m NaCl (S9888, Sigma–Aldrich, USA), 0.01%–0.1% Tween‐20, DEPC‐water, pH 7.5. Streptavidin Beads were mixed with cell lysis, and incubated overnight at 4 °C, followed by three washes with pre‐cooled lysis buffer. Products attached to the magnetic beads were boiled in lysis buffer and SDS loading buffer for 10 min and analyzed by western blotting.

### Plasmid Construction and siRNA Synthesis

Flag‐tag SPTBN1 wild‐type pcDNA3.1 plasmid, Flag‐tag SPTBN1 CH1‐2 pcDNA3.1 plasmid, Flag‐tag SPTBN1 spe1‐5 pcDNA3.1 plasmid, Flag‐tag SPTBN1 spe6‐10 pcDNA3.1 plasmid, Flag‐tag SPTBN1 spe11‐17 pcDNA3.1 plasmid, Flag‐tag SPTBN1 C domain pcDNA3.1 plasmid, Wnt3a expression pDNA3.1 plasmid, Dicer1 expression pCMV3 plasmid, LEF1 active luciferase report pLG3 plasmid, and Renille luciferase report pRL‐TK plasmid were constructed with assistance from HZREPOBIO, China. The maps and sequences are shown in Appendix SB (Supporting Information) plasmids. Gene sequences were amplified, purified, and ligated into vectors, which were transformed into DH5α (C502‐02, Vazyme, China) and spread onto LB Agar Plates at 37 °C overnight. Colonies were screened and sequenced for correct insertion. Positive plasmids were extracted by using the FastPure EndoFree Plasmid Maxi Kit (DC202‐01, Vazyme, China). The sequences of siRNAs for P120‐catenin and α‐catenin knockdown are shown in Appendix SA Table S2 (Supporting Information).

### Immunofluorescence

Pretreated cells were fixed with 4% paraformaldehyde (FD3628, Fudebio, China) for 20 min, then incubated in 1% Triton X‐100 (FD9128, Fudebio, China) in PBS at 4 °C for 10 min. Samples were blocked with immunostaining Block Buffer (PR30008, Proteintech, China) at 37 °C for 30 min, incubated with primary antibody at 4 °C overnight, followed by incubation with fluorescently labeled secondary antibodies at 37 °C for 1 h. Samples were observed with a confocal laser scanning microscope (FV3000, Olympus, Japan). The antibodies used in this study are shown in Appendix SA Table S1 (Supporting Information).

### Immunocytochemistry

Slides from HCC in situ mice were heated at 50 °C for 15 min to remove paraffin in Gemini AS (Thermo Scientific, USA) and immersed in xylene. The slides were passed through decreasing concentrations of alcohol (100%, 95%, and 70%) and rinsed in water to rehydrate the tissue. Slides were heated in citrate‐EDTA Solution (abs9249, Absin, China) for antigen retrieval and incubated with block buffer. Slides were incubated for the primary antibody at 4 °C overnight and with the secondary antibody at room temperature for 1 h. The slides were treated with M&R HRP/DAB Detection IHC Kit (HC301‐01, Vazyme, China) and scanned with VS200 (Olympus, Japan). The antibodies used in this study are shown in Appendix SA Table S1 (Supporting Information).

### Quantitative Reverse Transcription PCR (qRT‐PCR) and Sanger Sequencing

Total RNAs from the tissues or HCC cells were extracted and purified using the RNA‐Quick purification kit (RN001, esunbio, China) according to the manufacturer's manual. Small RNA reverse transcription was performed using stem‐loop reverse transcription primers according to the instruction of riboSCRIPT Reverse Transcription Kit (C11027, Ribobio, China). mRNA reverse transcription was carried out using ABScript Neo RT Master Mix (RK20433, Abclonal, RK20433). PCR amplification was conducted at 95 °C for 15 s and 60 °C for 30 s, using 2X Universal SYBR Green Fast qPCR Mix (RK20433, Abclonal, China). U6 (Bulge‐Loop U6 qPCR Primer, MQPS0000002, Ribobio, China) and β‐actin were used as normalization controls for sRNAs and mRNAs, respectively. Sanger sequencing of purified amplification products was performed using the BigDye Teminator V3.1 Cycle Sequencing Kit (Applied Biosystems, USA) with a 3730×L DNA analyzer (Applied Biosystems, USA). The primers used in the study are shown in Appendix SA Table S3 (Supporting Information).

### Cell Culture, Transfection, and Treatment

As previously described, SNU‐449 cells were cultured in RPMI 1640 medium (GIBCO, USA). LM3 cells were cultured in Dulbecco's modified Eagle's medium (Thermo Fisher Scientific, USA). Both media were supplemented with 10% fetal bovine serum (FBS) (BioIND, China). The cells were cultured in a humidified incubator at 37 °C with 5% carbon dioxide (CO_2_). Following plating into 6‐well plates overnight, HCC cells were transfected with the constructed small RNAs or plasmids using lipofectamine 3000 (L3000015, Thermo Fisher Scientific, USA), according to the manufacturer's manual. The cells were treated with MG132 (10 µm, HY‐13259, MCE, USA), Salinomycin (1 µm, HY‐15597, MCE, USA), Dalosirvat (20 nm, SM‐04554, MCE, USA), Kallistatin (5nm, HY‐P71143, MCE, USA), and Wnt3a in medium, as required by the experimental protocol.

### Cell Counting Kit‐8 (CCK‐8)

Cell proliferation was assessed using the CCK‐8 assay. SNU‐449 and LM3 cell lines transfected with HCETSR or negative controls were seeded into 96‐well plates and cultured for 4 days. Cells were incubated with CCK‐8 reagent (CK04‐11, Dojindo Molecular Technologies, Japan) for 1 h (1:10 dilution), and absorbance at 450 nm was measured by a spectrophotometric reader (Multiskan FC, Thermo Fisher Scientific, USA).

### Transwell Assay

The transwell assay with Matrigel was employed to evaluate the invasiveness of the HCC cells. HCC cells transfected with HCETSR or negative control for 24 h were incubated in serum‐free medium for 2 h in the upper chamber (3422, Corning, USA) pretreated with Matrigel (356234, BD Biosciences, USA). Cells were resuspended in the upper chamber with serum‐free medium, while the lower chamber contained medium with 20% serum. After 24 h of culture, cells were fixed with paraformaldehyde (FD3628, Fudebio, China), stained with crystal violet (HY‐B0324A, MCE, USA), and counted to assess invasive capacity.

### Dual‐Luciferase Reporter Assay

As previously described,^[^ ^71^ ^]^ the LEF1 luciferase reporter plasmid was synthesized and cloned into pLG3 vector with the assistance of REPOBIO (Hangzhou, China). The constructed vector was co‐transfected into HCC cells with α‐catenin siRNAs or P120‐catenin siRNAs using lipofectamine 3000 (L3000015, Thermo Fisher Scientific, USA). After 48 h, dual luciferase activity was measured using the dual‐luciferase reporter assay kit (DL101, Vazyme, China) according to the manufacturer's instructions.

### Tumor Model

BALB/c male nude mice (6–8 weeks old) were provided by the Zhejiang Academy of Medical Sciences and maintained according to the standard of the National Institute for the Protection and Use of Laboratory Animals Guidelines. The animal research protocol was approved by the Institutional Animal Care and Use Committee, Zhejiang Center of Laboratory Animals (ZJCLA‐IACUC‐20010183). After anesthesia, surgical procedures were performed for liver implantation of LM3 luciferase‐expressing cells. One week later, luciferase substrate (HY‐12591B, MCE, USA) was injected intraperitoneally into the mice, followed by observation and grouping based on fluorescence signals. Every 3 days, two groups were injected with 10 nmol of HCETSR agomir analog (SunYa, China) or negative control, for a total of five injections. After 4 weeks, all mice were euthanized by CO_2_ inhalation. Lung and celiac lymph node metastases were evaluated using live bioluminescence imaging.

### Patients and Specimens

HCC tissues and paired adjacent non‐tumor tissues were obtained from patients who underwent hepatectomy and were diagnosed as HCC at the First Affiliated Hospital, College of Medicine, Zhejiang University, China. The histological stage of HCC patients was according to the 8th edition of the American Joint Committee on Cancer; tumor, node, metastasis (AJCC‐TNM) classification.^[^ ^72^ ^]^ This research protocol was approved by the Research Ethical Committee of the First Affiliated Hospital, School of Medicine, Zhejiang University (2018‐768). The participants provided written informed consent to participate in this study. A total of 126 HCC patients were enrolled in this study. 48(38.1%) female and 78(61.9%) male. Sixty‐one (48.8%) were alpha‐fetoprotein (AFP) positive (> 20 ng mL^−1^), and 64 (50.8%) were AFP negative. One hundred two (81.0%) were positive hepatitis B virus (HBV) infection, and 21 (16.7%) were negative for HBV infection. All patient characteristics are shown in Appendix SA Table S4 (Supporting Information).

### Bioinformation Analysis and Gene‐Pathway Correlation Analysis

As previously described,^[^ ^71^ ^]^ Kyoto Encyclopedia of Genes and Genomes (KEGG) and Gene Ontology (GO) analysis of the differential genes were performed with the online database KEGG Orthology Based Annotation System (KOBAS) 3.0 (http://bioinfo.org/kobas). The differentially expressed genes from five pathways were screened and constructed as gene sets. The networks between downstream of LEF1 and the gene sets were constructed using the Mantel test based on the Bray–Curtis distance.

### Statistical Analysis

Quantitative variables were reported as mean ± standard deviation (SD) or median ± interquartile range (IQR). Student's *t*‐test or Mann‐Whitney test was used to compare quantitative variables between two groups. For comparisons involving more than two groups, analysis of variance (ANOVA) followed by Newman–Keuls post‐hoc tests was conducted. Categorical variables were compared using the chi‐square test or Fisher's exact test. Relative expression levels of HCETSR, tRNA‐Glu/TTC, pre‐tRNA‐Glu, MMP7, and CCDN1 were assessed using ΔCt values. U6 and β‐actin were used as normalization controls for sRNAs and mRNAs, respectively. The optimal cut‐off value for HCETSR expression of 126 HCC samples was determined using the receiver operating characteristic (ROC) curve by calculating the best Youden index (sensitivity + specificity – 1). The prognostic prediction value of HCETSR in HCC was evaluated through the area under the receiver operating characteristic (AUROC) curve. Kaplan–Meier survival curves were analyzed using the log‐rank test. Multivariate Cox proportional hazards regression analysis was used to identify risk factors for HCC prognosis. All experiments were independently repeated at least three times. Statistical analyses were performed using Statistical Product and Service Solutions (SPSS) software (Version 19.0, IBM, USA). All tests were two‐tailed, with a significance threshold set at *p* < 0.05. Significance levels were denoted as ^*^, ^**^, ^***^, and ^****^ corresponding to p values of < 0.05, < 0.01, < 0.001, and < 0.0001, respectively.

### Ethics Approval and Consent to Participate

The studies involving human participants were reviewed and approved by the Ethical Committee of the First Affiliated Hospital, Zhejiang University School of Medicine, in accordance with the Declaration of Helsinki (2018‐768). The patients/participants provided their written informed consent to participate in this study. The animal study was reviewed and approved by the Institutional Animal Care and Use Committee (IACUC), Zhejiang Center of Laboratory Animals (ZJCLA) (ZJCLA‐IACUC‐20010183).

## Conflict of Interest

The authors declare no conflict of interest.

## Author Contributions

The project was designed and supervised by X.X. and T.R. T.R., K.Z., Z.M., J.W., Y.P., Q.Y., C.C., A.X., J.G., N.T., and J.Z. conducted the experiment. T.R. and K.Z. completed animal study. All authors contributed to the data analysis. T.R., S.Z., J.L., and X.X. prepared the manuscript. All authors have read and approved the final manuscript.

## Supporting information



Supplemental Appendix A

Supplemental Appendix B

Supplemental Appendix C

Supplemental Appendix D

Supplemental Appendix E

Supplemental Appendix F

Supplemental Appendix G

Supplemental Appendix H

## Data Availability

The data that support the findings of this study are available from the corresponding author upon reasonable request.
