# Supplementary material for: A Novel tRF, HCETSR, Derived From tRNA‐Glu/TTC, Inhibits HCC Malignancy by Regulating the SPBTN1‐catenin Complex Axis
Source: Adv Sci (Weinh). 2025 Feb 8;12(13):2415229. doi: 10.1002/advs.202415229 (PMC11967833; doi:10.1002/advs.202415229)

**Appendix A Table1. Antibodies used in this study.**

| Antibody | Manufacturer | Code |
| --- | --- | --- |
| SPTBN1-Specific Polyclonal antibody | Proteintech | 19722-1-AP |
| Beta Catenin Monoclonal antibody | Proteintech | 66379-1-Ig |
| Active β-Catenin Rabbit mAb | ABclonal | A22180 |
| α-Catenin Rabbit pAb | ABclonal | A5635 |
| P120 Catenin Polyclonal antibody | Proteintech | 12180-1-AP |
| APC Rabbit mAb | ABclonal | A17912 |
| Axin1 (C76H11) Rabbit mAb | Cell Signaling Technology | 2087 |
| Phospho-β-Catenin-S45 Rabbit pAb | ABclonal | AP0580) |
| P-β-atenin-S29/33/37/T41 Rabbit pAb | ABclonal | AP1076 |
| P-GSK3β-Y216 GSK3α-Y279 Rabbit pAb | ABclonal | AP0261 |
| GSK3β Rabbit pAb | ABclonal | A2081 |
| LEF1 Rabbit pAb | ABclonal | A0909 |
| PSMA7 Polyclonal antibody | Proteintech | 15219 |
| MMP7 Rabbit pAb | ABclonal | A0695 |
| Cyclin D1 (CCDN1) Rabbit mAb | ABclonal | A19038 |
| SNRNP70 Rabbit mAb | ABclonal | A24073 |
| U2AF1 Rabbit pAb | ABclonal | A13166 |
| U2AF2 Rabbit pAb | ABclonal | A4552 |
| PRPF4 Rabbit pAb | ABclonal | A21132 |
| PRPF8 Rabbit pAb | ABclonal | A4575 |
| DDX5 Rabbit pAb | ABclonal | A11339 |
| EFTUD2 Rabbit pAb | ABclonal | A7040 |
| PRP19 Rabbit pAb | ABclonal | A9660 |
| CDC5L Rabbit pAb | ABclonal | A9527 |
| Hsc70/HSPA8 Rabbit mAb | ABclonal | A0415 |
| DICER1 Polyclonal antibody | Proteintech | 20567-1-AP |
| Mouse anti DDDDK-Tag mAb | ABclonal | AE005 |
| Ubiquitin rabbit | Proteintech | 10201-2-AP |
| Rabbit negative IgG | ABclonal | AC005 |
| Rabbit IgG | Cell Signaling Technology | 2729 |
| Anti-rabbit IgG, HRP-linked Antibody | Cell Signaling Technology | 7074 |
| Anti-mouse IgG, HRP-linked Antibody | Cell Signaling Technology | 7076 |
| β-Actin Rabbit mAb (HRP Conjugate) | Cell Signaling Technology | 5125 |
| TBP Rabbit pAb | ABclonal | A2192 |
| FITC- Goat Anti-Rat IgG (H+L) | Jackson ImmunoResearch | 112-096-003 |
| Cy3- IgG Goat Anti-Rat IgG (H+L) | Jackson ImmunoResearch | 112-166-003 |
| FITC- Goat Anti-Mouse IgG (H+L) | Jackson ImmunoResearch | 115-096-003 |
| Cy3-Goat anti-Mouse IgG | Jackson ImmunoResearch | 115-166-003 |

**Appendix A Table2. The sequences of siRNAs**

| Name | Sequence |
| --- | --- |
| si-P120 cantenin-1-S | CGCCACUAUGAAGAUGGUUAUTT |
| si-P120 cantenin-1-A | AUAACCAUCUUCAUAGUGGCGTT |
| si-P120 cantenin-2-S | CUCCCAAUGUUGCCAACAAUATT |
| si-P120 cantenin-2-A | UAUUGUUGGCAACAUUGGGAGTT |
| si-α-cantenin-1-S | CCCUCUGUCCUCAGGUUAUUATT |
| si-α-cantenin-1-A | UAAUAACCUGAGGACAGAGGGTT |
| si-α-cantenin-2-S | CCUCAGAGAUGGACAACUAUGTT |
| si-α-cantenin-2-A | CAUAGUUGUCCAUCUCUGAGGTT |

**Appendix A Table3.** **The primers sequences used in this study**

| Name | Sequence |
| --- | --- |
| CCND1-F | GATGCCAACCTCCTCAACGA |
| CCND1-R | GGAAGCGGTCCAGGTAGTTC |
| CTNNB1-F | CATCTACACAGTTTGATGCTGCT |
| CTNNB1-R | GCAGTTTTGTCAGTTCAGGGA |
| MMP7-F | CATGATTGGCTTTGCGCGAG |
| MMP7-R | ATCTCCTCCGAGACCTGTCC |
| HCETSR-RT | GTCGTATCCAGTGCAGGGTCCGAGGTATTCGCACTGGATACGACCCAGGAA |
| HCETSR-F | TCCCATATGGTCTAGCGGTTA |
| tRNA-Glu-TTC-RT | GTCGTATCCAGTGCAGGGTCCGAGGTGCACTGGATACGACTCGAAC |
| tRNA-Glu-TTC-F | TATGGTCTAGCGGTTAGGATT |
| Pre-tRNA-Glu-TTC-(chr2)RT | GTCGTATCCAGTGCAGGGTCCGAGGTGCACTGGATACGAC*GCCACTT* |
| Pre-tRNA-Glu-TTC-(chr13)RT | GTCGTATCCAGTGCAGGGTCCGAGGTGCACTGGATACGAC*TAAGGAA* |
| Rverser primer | CAGTGCAGGGTCCGAGGTGC |

**Appendix A Table4: Patient characteristics**

| Characteristics |  |
| --- | --- |
| Age (yr) |  |
| ≤ 65 | 34 (27.0%) |
| > 65 | 92 (73.0%) |
| Gender, n (%) |  |
| Male | 78 (61.9%) |
| Female | 48 (38.1%) |
| HBV, n (%) |  |
| Negative | 21 (16.7%) |
| Positive | 102 (81.0%) |
| No detected | 3 (2.4%) |
| AFP, n (%) |  |
| ≤ 20 ng/ml | 64 (50.8%) |
| > 20 ng/ml | 61 (48.4%) |
| No detected | 1 (0.8%) |
| AFP, n (%) |  |
| ≤ 100 ng/ml | 58 (46.0%) |
| > 100 ng/ml | 67 (53.2%) |
| No detected | 1 (0.8%) |
| AFP, n (%) |  |
| ≤ 400 ng/ml | 77 (61.1%) |
| > 400 ng/ml | 48 (38.1%) |
| No detected | 1 (0.8%) |
| Tumour size, n (%) |  |
| ≤ 5 cm | 33 (26.2%) |
| > 5 cm | 93 (73.8%) |
| T Stage, n (%) |  |
| I | 53 (42.1%) |
| II | 31 (24.6%) |
| III | 13 (10.3%) |
| IV | 29 (23.0%) |
| AJCC Stage, n (%) |  |
| I | 44 (34.9%) |
| II | 22 (17.5%) |
| III | 49 (38.9%) |
| IV | 11 (8.7%) |
| Tumour number, n (%) |  |
| Isolated | 83 (65.9%) |
| multiple | 43 (34.1%) |
| Macrovascular invasion, n (%) |  |
| Negative | 88 (65.8%) |
| Positive | 38 (34.1%) |
| Albumin (g/L) | 43.39 ± 4.13 |
| Prothrombin time (s) | 11.93 ± 1.22 |
| Creatinine | 70.80 ± 12.62 |
| Alanine aminotransferase (U/L) | 35.29 ± 20.89 |
| Aspartate aminotransferase (U/L) | 43.87 ± 28.54 |
| International normalized ratio | 1.04 ± 0.11 |
| Hemoglobin (g/L) | 142.98 ± 16.30 |
| Platelet (10^9^/L) | 175.80 ± 58.18 |
| Leukocyte (10^9^/L) | 5.78 ± 2.03 |

**Appendix A Figure1.**


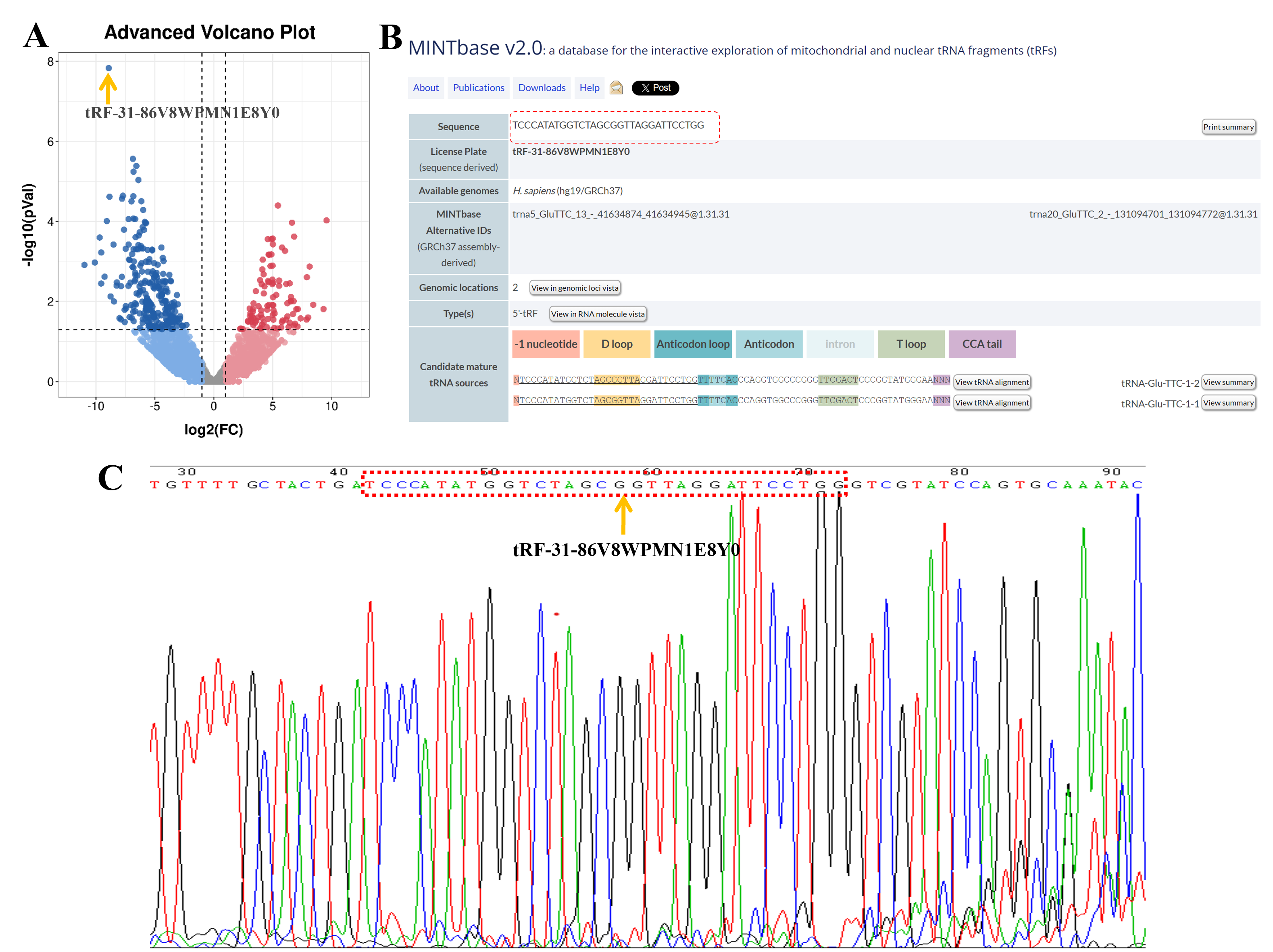


Appendix A Figure1: **A:** The volcano plot illustrates the expression levels of tRNA-derived fragments (tRFs) in hepatocellular carcinoma (HCC) compared to adjacent liver tissues. Notably, tRF-31-86V8WPMN1E8Y0 (HCETSR) demonstrates the most significant differential expression. **B:** The sequence of tRF-31-86V8WPMN1E8Y0, as retrieved from MINTbase V2.0. **C:** Sanger sequencing confirms that the sequence of tRF-31-86V8WPMN1E8Y0 matches the sequence provided by MINTbase.

**Appendix A Figure2.**


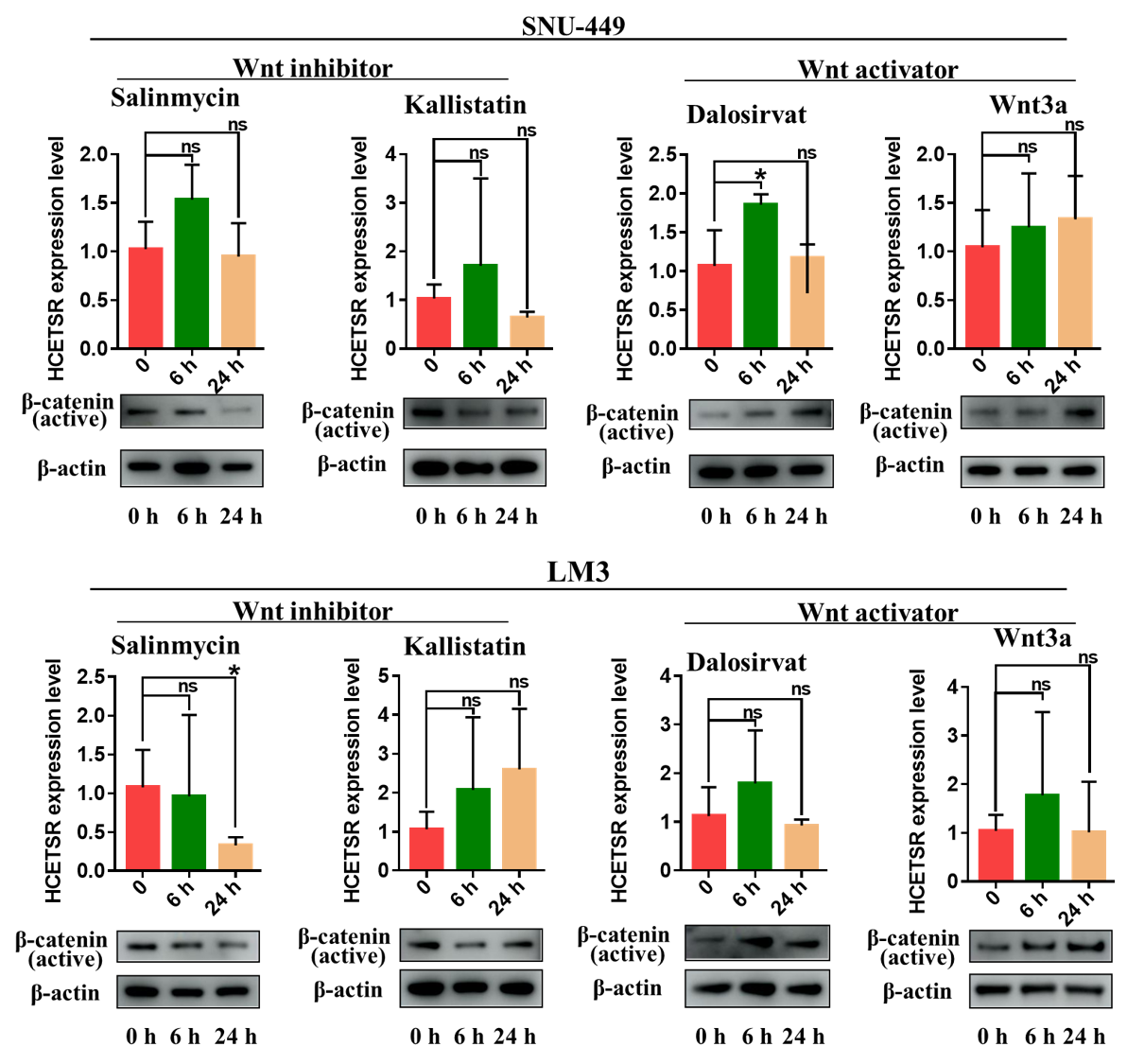


Appendix A Figure2: The expression levels of HCETSR and active β-catenin in SNU-449 and LM3 cells following treatment with two Wnt inhibitors (salinomycin and kallistatin) and two Wnt activators (Wnt3a and dalosirvat).

**Appendix A Figure3.**

We attempted to knockout HCETSR using the CRISPR-Cas9 system. Considering that HCETSR may originate from tRNA-Glu/TTC on chromosome 2 and tRNA-Glu/TTC on chromosome 13, two vectors were designed, each expressing two guide RNAs (gRNAs). These vectors, along with the Cas9 component, were co-transfected into SNU-449 and LM3 cells. A significant number of cells exhibited cell death two days post-transfection. Subsequent selection and identification revealed that the knockout was unsuccessful. The knockout of tRNA-Glu/TTC is lethal to HCC cells.

2 gRNA sequencing for targeting to tRNA-Glu/TTC chr2:

CCATACCGGGAGTCGAACCC TCCTAACCGCTAGACCATAT

2 gRNA sequencing for targeting to tRNA-Glu/TTC chr13:

CCATACCGGGAGTCGAACCC CAGCTTCAGTAGCGCAGAGG


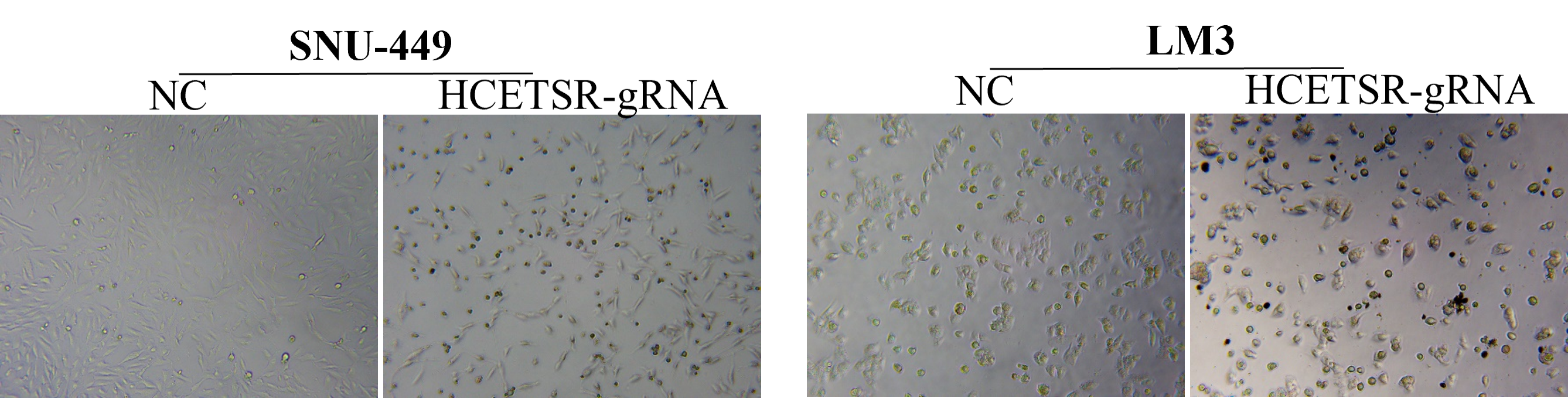

Supplement: Supplementary file 1 — Supplemental Appendix A [file ADVS-12-2415229-s003.docx]
